# Radio-Frequency Safety Assessment of Stents in Blood Vessels During Magnetic Resonance Imaging

**DOI:** 10.3389/fphys.2018.01439

**Published:** 2018-10-22

**Authors:** Kyoko Fujimoto, Leonardo M. Angelone, Elena Lucano, Sunder S. Rajan, Maria Ida Iacono

**Affiliations:** Division of Biomedical Physics, Office of Science and Engineering Laboratory, Center for Devices and Radiological Health, U.S. Food and Drug Administration, Silver Spring, MD, United States

**Keywords:** RF safety, SAR, blood vessels, small implantable device, tangential electric field

## Abstract

**Purpose:** The purpose of this study was to investigate the need for high-resolution detailed anatomical modeling to correctly estimate radio-frequency (RF) safety during magnetic resonance imaging (MRI). RF-induced heating near metallic implanted devices depends on the electric field tangential to the device (*E_tan_*). *E_tan_* and specific absorption rate (SAR) were analyzed in blood vessels of an anatomical model to understand if a standard gel phantom accurately represents the potential heating in tissues due to passive vascular implants such as stents.

**Methods:** A numerical model of an RF birdcage body coil and an anatomically realistic virtual patient with a native spatial resolution of 1 mm^3^ were used to simulate the *in vivo* electric field at 64 MHz (1.5 T MRI system). Maximum values of SAR inside the blood vessels were calculated and compared with peaks in a numerical model of the ASTM gel phantom to see if the results from the simplified and homogeneous gel phantom were comparable to the results from the anatomical model. *E_tan_* values were also calculated in selected stent trajectories inside blood vessels and compared with the ASTM result.

**Results:** Peak SAR values in blood vessels were up to ten times higher than those found in the ASTM standard gel phantom. Peaks were found in clinically significant anatomical locations, where stents are implanted as per intended use. Furthermore, *E_tan_* results showed that volume-averaged SAR values might not be sufficient to assess RF safety.

**Conclusion:** Computational modeling with a high-resolution anatomical model indicated higher values of the incident electric field compared to the standard testing approach. Further investigation will help develop a robust safety testing method which reflects clinically realistic conditions.

## Introduction

Magnetic resonance imaging (MRI) has become one of the most popular medical imaging modalities thanks to its noninvasive nature based on non-ionizing radiation. It is often used as the diagnostic tool of choice for various diseases and represents a safe imaging device, although some risks still exist and need to be mitigated. For example, the radio-frequency (RF) power used to elicit the magnetic resonance (MR) signal may induce a temperature increase inside the patient’s body. Furthermore, implantable medical devices with electrically conductive components may act as RF antennas resulting in amplified energy absorption and temperature increase near the device, which may cause possible thermal tissue injury for the patient.

The ASTM F2182-11 standard proposes a testing method to assess MR RF heating safety of passive implantable devices such as stents (ASTM F2182-11a, 2011). The standard provides indication on how to evaluate the whole-body averaged specific absorption rate (SAR), the local SAR, and the temperature rise caused by the device using a rectangular box filled with a saline gel (“ASTM phantom”).

The results of the ASTM testing are then used to generate the MR Conditional labeling of the device. Historically, ASTM testing has been considered to represent an upper limit for *in vivo* heating. Although the ASTM phantom allows heating assessment under well controlled conditions, one open question is how well an electrically homogeneous model can represent a whole human body with respect to RF-induced heating for implants. Anatomical structures in the human body are highly intricate and inhomogeneous. In addition, ASTM testing is performed with the phantom placed at the center of the RF coil whereas patients can be scanned in multiple positions depending on the anatomical area under clinical examination. Therefore, RF-induced heating measurements due to an implanted device performed using the ASTM phantom may not necessarily be the worst case and may not be directly translated into *in vivo* scenarios.

Choosing the right phantom to obtain a realistic assessment of *in vivo* heating in the human body is challenging. Adams et al. (2005) used an *ex vivo* superficial femoral artery to test 15 stents. It is difficult to perform such experiment for every possible stent because of the wide variety of shapes, materials (O’Brien and Carroll, 2009), and types, such as drug-eluting stents (Shellock and Forder, 2005; Garg and Serruys, 2010). Hug et al. (2000) tested 19 stents inside of yogurt, which has the MR relaxation time close to human myocardial tissue. Their choice of material successfully enabled them to observe safety of different stents including RF heating in the synthesized conditions. However, there remains an open question about the interactions of stents with the surrounding tissues such as muscle and fat. Creating an anthropomorphic phantom is difficult because tissue-mimicking medium and intricate structures of a human body are very challenging to fabricate.

Computational modeling is often used for medical device RF safety evaluation as a complementary approach to experimental measurements. The finite-difference time-domain (FDTD) method is one of the most popular techniques used to assess RF safety, as it allows calculating electromagnetic fields in anatomically detailed numerical whole-body human models. Currently available whole-body anatomical models have up to 77 anatomical structures (Ackerman, 1998; Nagaoka et al., 2003; Christ et al., 2009; Massey and Yilmaz, 2016). Some of these models have up to 1 mm spatial resolution and a fine segmentation of blood vessels, which are ideal for the MR RF safety analysis of a stent. Despite the availability of such high-resolution models, previous studies did not exploit the highest resolution of the models due to computational resource limitations.

For example, Homann et al. (2011) showed that a spatial resolution of 5 mm is sufficient to simulate the SAR of five volunteers. However, 5 mm spatial resolution does not allow the discernment and characterization of very thin structures such as blood vessels. With the advancement of computational hardware resources today, such as the amount of random-access memory (RAM) and the availability of a graphic processing unit (GPU), FDTD-based models up to 1 Giga cells – corresponding to a whole body spatial resolution of less than 2 mm – can now be handled. Such detailed simulations may provide more accurate predictions of the specific source of RF-induced heating and help us identify devices that are at higher risk for RF heating, due to their anatomical location.

In addition, using local SAR as the only parameter to assess RF-induced heating presents some significant limitations. Indeed, local SAR might not correlate directly with heating as it results from all the components of the electric field, while the tangential component of the electric field is exclusively responsible for the currents induced in the implant. As such, the local background SAR (tissue SAR without a device) may not be the best parameter to study RF-induced heating compared to tangential electric field.

In this study, we evaluated the local background voxel-averaged SAR (*SAR_raw_*), 1 g-averaged SAR (*SAR*_1_*_g_*), and 10 g-averaged SAR (*SAR*_10_*_g_*) in the vascular territories of the 1 mm isotropic numerical whole-body AustinMan human model and in the ASTM phantom. The tangential electric field (*E_tan_*) was also studied for selected trajectories typical of stents location within the vessels. Four imaging landmark positions of the human model within the RF coil were simulated with a local numerical resolution up to 0.98 mm.

## Materials and Methods

### Computational Modeling Setup

Electromagnetic field simulations at 64 MHz (1.5 T MRI system) were performed using the commercially available FDTD platform, Sim4Life (Zurich Med Tech, Switzerland). The birdcage coil was modeled based on the MITS1.5 physical system (Zurich Med Tech, Zurich, Switzerland). As described by Lucano et al. (2016), the coil was 750 mm in length and 650 mm in diameter, included 16 legs and two rings. All the coil materials were modeled as perfect electric conductors (PEC). The coil was driven in quadrature mode with two sources of equal amplitude with a 90°phase shift. The electrical components (1 kΩ resistors and 69.5 pF capacitors) were distributed in parallel on the rings. The MRI RF exposure was modeled as a continuous wave, which is the typical approach used in literature. It is not directly scalable to the exposures at the clinical MRI scanners; however, it provides a worst-case exposure scenario as the clinical MR sequences never reach a 100% duty cycle.

The high-resolution AustinMan v2.4 model (Massey and Yilmaz, 2016) with 1 mm isotropic spatial resolution was chosen because the vascular territories were segmented in detail although some of the very thin vessels are not continuous. The model has 64 anatomical structures. Each structure was assigned to a material with a specific electrical conductivity, permittivity, and mass density based on Gabriel (1996).

### Numerical Implementation

A multi-grid FDTD approach was used to discretize the simulation domain. In the multi-grid approach, a region with a main grid and another with a fine local grid were generated. The main grid with 2 mm isotropic resolution was used to discretize the anatomy of the patient. The fine local grid was used to discretize the fine components of the coil. As a result, some portions of the AustinMan model were discretized up to 0.98 mm in each dimension. A maximum isotropic resolution of 2 mm was used for the main grid as this was the highest achievable resolution utilizing all the available computational resources on our workstation (128 GB of RAM and NVIDIA Tesla K80 GPU).

### SAR Values in ASTM vs. AustinMan

Four simulations were performed with the AustinMan model at four different imaging landmarks with the isocenter of the coil placed at the brain, the heart, the hip bone, and the knee. These body parts are scanned often with an MRI for diagnostic purposes. An additional simulation was run using the ASTM phantom model at the isocenter of the coil. The conductivity of the gel was 0.47 S/m. Each simulation with the AustinMan model took approximately 27 h. The simulation with the ASTM phantom took approximately 4 h.

The maximum *SARraw*, *SAR*_1_*_g_*, and *SAR*_10_*_g_* values were calculated over the entire ASTM phantom as well as within the 10 cm trajectory as recommended in the standard. The maximum SAR values were also calculated in the blood vessels of the AustinMan model at each landmark. 3D surface maps of the SAR distribution on the vessels were generated to observe the appearance of any localized high exposure. In addition, axial slices of SAR were compared with the anatomical segmentation to identify the vessels with high exposure.

### Electric Field Tangential to Stents in Blood Vessels

Five stent locations were chosen and studied from Shellock (2014) as well as the archives of the premarket submissions on the U.S. Food and Drug Administration website (U.S. Food and Drug Administration, 2018). The location, orientation, and length of each stent trajectory in the AustinMan model was determined to calculate the tangential electric field.

Five case studies were analyzed for the ascending aorta, the brachial artery, the femoral artery, the iliac artery, and the popliteal artery. Stent trajectories were created based on the centerline of each blood vessel to model realistic scenarios. The centerlines were calculated in MATLAB (The MathWorks, Inc., Natick, MA, United States) by binarizing the label map of a specific vessel and determining the centroid of the consecutive axial slices. The centerline was then imported into Sim4Life to create a smooth trajectory. The *E_tan_* value was calculated along each trajectory using the IMSAFE module in Sim4Life. The magnitudes of *E_tan_* values were calculated offline.

*E_tan_* values were also calculated in the gel of ASTM phantom. A straight 10 cm trajectory was placed in the gel where the electric field is high and homogeneous as recommended by ASTM standard (ASTM F2182-11a, 2011). The trajectory was placed along *z*-axis, 3.7 cm away from the wall of the container in *x*-axis, and the middle plane in *y*-axis. This satisfies the recommendation in the standard (at least 2 cm away from the gel surface, bottom, and walls of the container). Magnitudes of *E_tan_* were calculated by following the same procedure as explained above for the stent trajectories.

### Normalization

All the results were normalized to satisfy the limits of SAR defined for normal operating mode (IEC, 2015). Specifically, the normalization coefficient was calculated to ensure that the following three conditions were all satisfied: whole-body SAR ≤ 2 W/kg, head SAR ≤ 3.2 W/kg, and partial-body SAR ≤ 2–10 W/kg. The exact limit for partial-body SAR limit was determined based on the exposed mass covered under the RF coil at the landmark as follows.

$${\mathrm{SAR}}_{\mathrm{exposed}}=10W/\mathrm{kg}-\left(8W/\mathrm{kg}\times \frac{\mathrm{exposed mass of AustinMan}}{\mathrm{whole body mass of AustinMan}}\right)$$

In this study, the whole-body SAR of 2 W/kg was the limiting safety condition for all the simulations except for the simulation at the heart landmark, which was normalized using the partial-body SAR.

## Results

### SAR Values in ASTM vs. AustinMan

**FIGURE 1:**
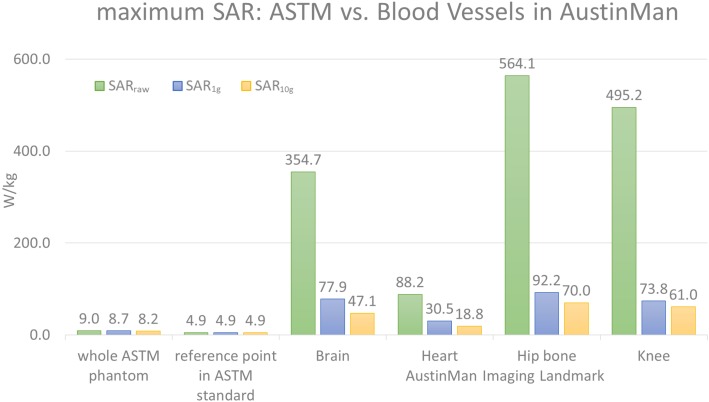
The maximum values of *SAR_raw_*, *SAR*_1_*_g_*, and *SAR*_10_*_g_* values are shown for the ASTM phantom model and the blood vessels at four different landmarks in the AustinMan model. The maximum SAR values in the ASTM phantom were identified in the whole ASTM phantom and within the 10 cm trajectory placed in the reference location suggested by the ASTM standard. The maximum SAR values in ASTM regardless of locations were much lower than that in the AustinMan model at any image landmarks. The maximum *SAR*_10_*_g_* value in the blood vessels at the hip bone landmark was 70 W/kg, which was also the highest *SAR*_10_*_g_* value among all the anatomical structures in the same simulation.

The maximum *SAR_raw_*, *SAR*_1_*_g_*, and *SAR*_10_*_g_* values in the ASTM phantom were compared against those in the AustinMan model at four landmarks. The results are summarized in Figure 1. The maximum *SAR_raw_* value along the 10 cm trajectory was 4.9 W/kg. The ratio of the peak value at that location to the average SAR value of the phantom (2 W/kg) was 2.45.

The blood vessels of the AustinMan model showed much higher SAR values compared to the SAR values in gel regardless of volume averaging: the maximum values in the blood vessels were up to 63 times higher for *SAR_raw_* (564.1 W/kg vs. 9.0 W/kg), up to 11 times higher for *SAR*_1_*_g_* (92.2 W/kg vs. 8.7 W/kg), and up to nine times higher for *SAR*_10_*_g_* (70 W/kg vs. 8.2 W/kg) compared to the entire ASTM phantom results. The maximum values in the blood vessels were up to 115 times higher for *SAR_raw_* (564.1 W/kg vs. 4.9 W/kg), up to 19 times higher for *SAR*_1_*_g_* (92.2 W/kg vs. 4.9 W/kg), and up to 14 times higher for *SAR*_10_*_g_* (70 W/kg vs. 4.9 W/kg) compared to the reference point in the ASTM phantom as the standard suggests.

For the brain landmark, all the maximum SAR values were found in the heart region where various coronal stents can be implanted. For the heart landmark, the maximum SAR values were found at the different locations. The maximum *SAR_raw_* was located close to the skin where the AustinMan’s left elbow is rested on the oblique abdominal region. The maximum *SAR*_1_*_g_* was found in the AustinMan’s right oblique abdominal region. The maximum *SAR*_10_*_g_* was located in the lower spine where inferior and superior mesenteric stents can be implanted. At the hip bone landmark, all the maximum SAR values were found at the groin region where the brachytherapy devices can be inserted. These devices require MRI scans to calculate the dosage after the device is inserted in the body. For the knee landmark, the maximum *SAR_raw_* was at the left ankle where bypass grafts can be implanted. The maximum *SAR*_1_*_g_* and *SAR*_10_*_g_* were at the location where popliteal stents can be implanted.

### SAR Values in the Blood Vessels of AustinMan

**FIGURE 2:**
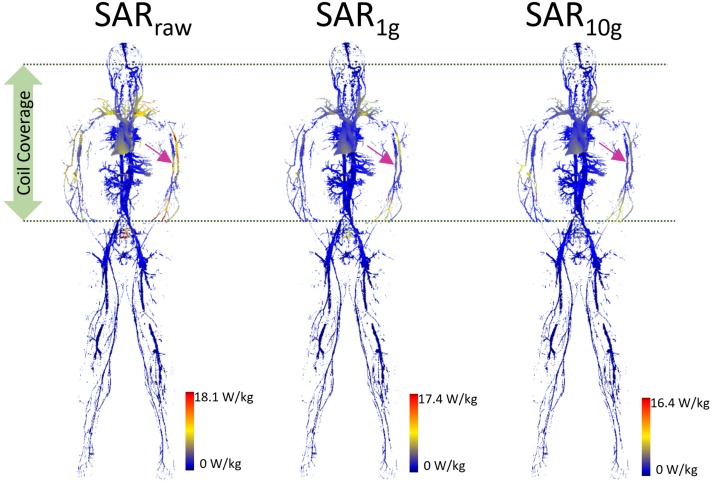
The blood vessel 3D SAR maps at the heart landmark are shown. The color scale was set such that the maximum was twice the peak SAR calculated in the whole ASTM phantom model (9.0, 8.7, and 8.2 W/kg for *SAR_raw_*, *SAR*_1_*_g_*, and *SAR*_10_*_g_*, respectively) to optimize the visualization of the peaks across all the models. The pink arrows pointing to the brachial artery show the example of high SAR values in *SAR_raw_* which were washed away in the *SAR*_1_*_g_* and *SAR*_10_*_g_* maps due to volume averaging.

The SAR maps from the AustinMan simulations were masked to evaluate the results exclusively in the blood vessels as shown in Figure 2. We performed masking after calculating volume-averaged SAR values. Thus, the SAR values of surrounding structures of blood vessels were considered in calculating over 1 and 10 g volumes. Clusters of high SAR values exceeding the ASTM peak were found for all *SAR_raw_*, *SAR*_1_*_g_*, and *SAR*_10_*_g_* results. In addition, there were some high SAR regions being washed out by volume averaging. For example, the brachial artery showed high *SAR_raw_*, but low *SAR*_1_*_g_* and *SAR*_10_*_g_* at the heart landmark (pink arrows in Figure 2). The SAR maps of the blood vessels at all the imaging landmarks (brain, heart, hip bone, and knee) are shown in Supplementary Figure S1.

**FIGURE 3:**
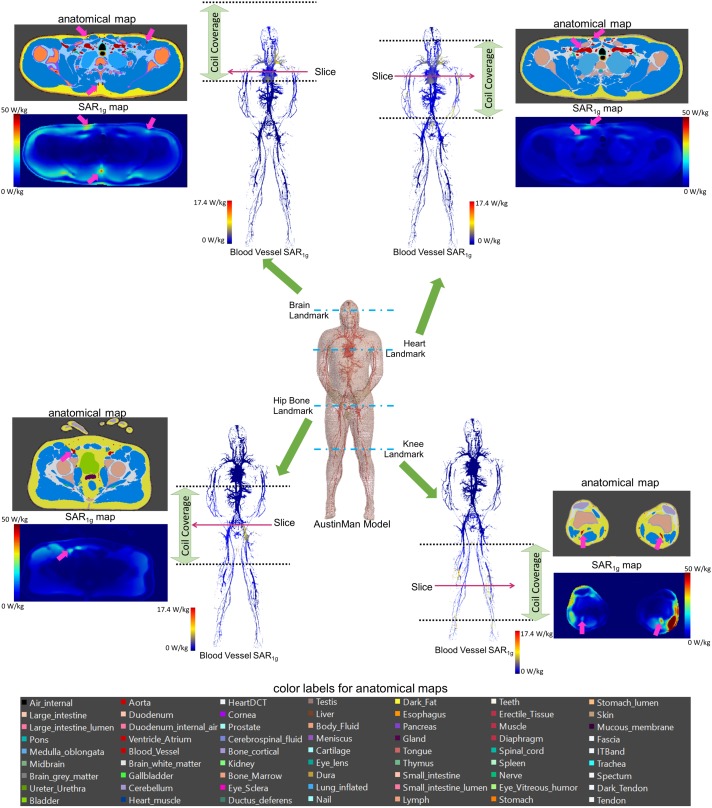
3D *SAR*_1_*_g_* maps in blood vessels are shown for all four landmarks. High *SAR*_1_*_g_* was reported in some of the blood vessels compared to surrounding tissues. Examples of high *SAR*_1_*_g_* in vessels are provided in axial views along with the corresponding anatomical maps.

All the SAR results were compared with the anatomical map of the AustinMan model. The blood vessels showed higher SAR compared to the surrounding tissues. Examples of such cases are shown in the axial slices in Figure 3 along with the corresponding anatomical maps. Blood vessels are shown in cyan and the pink arrows indicate those with high *SAR*_1_*_g_*. Maximum *SAR*_1_*_g_* in the blood vessels shown in these axial slices are 32, 20, 23, and 40 W/kg, for the brain, heart, hip bone, and knee landmarks, respectively.

### Electric Field Tangential to Stents in Blood Vessels

**FIGURE 4:**
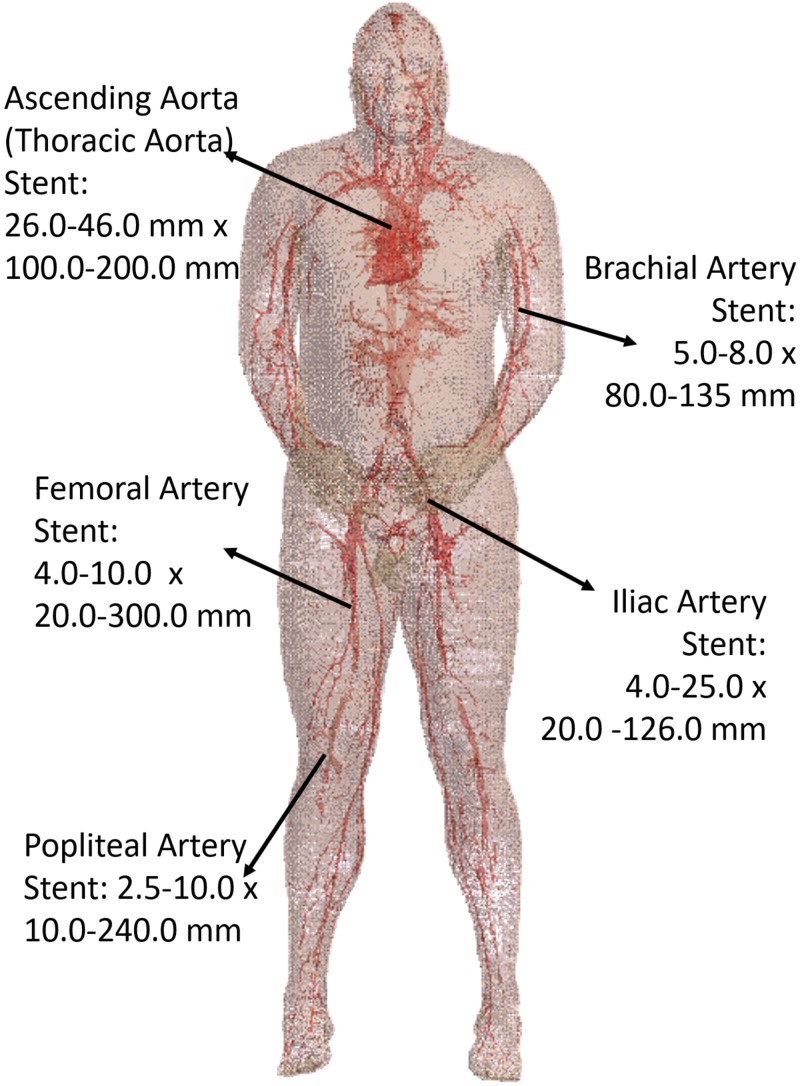
The blood vessels of the AustinMan model and its skin are shown. The black arrows indicate the five stent locations investigated. The lengths and diameters of commercially available stents are summarized. The blood vessels are modeled in detail although some discontinuities exist.

Five stent locations were identified in the AustinMan model (Figure 4). Stents for these selected locations can have a range of 10.0–300.0 mm in length and 2.5–46.0 mm in diameter. The lengths of the trajectories were 70.9 mm for the ascending aorta, 83.9 mm for the brachial artery, 179.7 mm for the femoral artery, 111.4 mm for the iliac artery, and 91.6 mm for the popliteal artery. Each length was determined based on the commercially available stents identified in Figure 4 as well as the vessel continuity of the AustinMan model.

**FIGURE 5:**
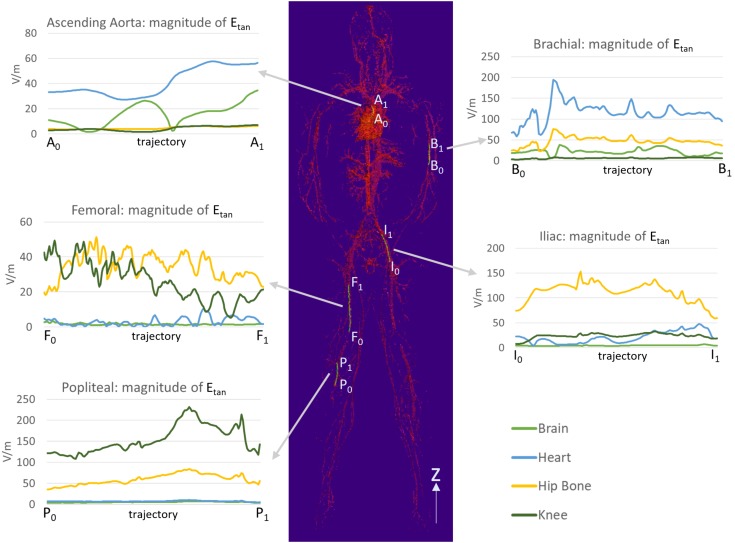
The magnitudes of *E_tan_* were calculated along five trajectories in the ascending aorta, the brachial artery, the femoral artery, the iliac artery, and the popliteal artery. The lengths of the trajectories were 70.9, 83.9, 179.7, 111.4, and 91.6 mm for the ascending aorta, the brachial artery, the femoral artery, the iliac artery, and the popliteal artery, respectively.

The *E_tan_* profile in each location is shown in Figure 5. The landmark highly affected the *E_tan_* values for all five different stent trajectories. High *E_tan_* values were found in the brachial artery (194.1 V/m) at the heart landmark, the iliac artery (153.2 V/m) at the hip bone landmark, and the popliteal artery (231.3 V/m) at the knee landmark. The *E_tan_* values were consistently high in the popliteal and iliac arteries at the hip bone landmark. The mean *E_tan_* value in the popliteal artery was 59.6 V/m and the mean *E_tan_* value at in the iliac artery was 111.5 V/m.

**FIGURE 6:**
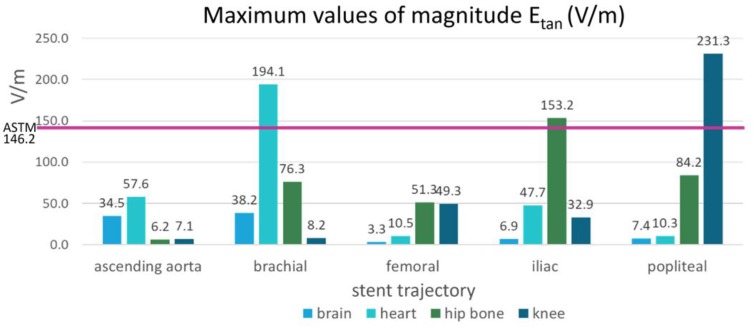
The maximum magnitudes of *E_tan_* calculated in the blood vessels of the five stent locations were compared with the maximum magnitude of *E_tan_* calculated in the ASTM phantom (shown by the pink horizontal line).

The maximum *E_tan_* values in five blood vessel locations and in the ASTM phantom were compared (Figure 6). The following three stent locations exceeded the maximum *E_tan_* in the gel of the ASTM phantom (146.2 V/m): the brachial artery at the heart landmark (194.1 V/m), the iliac artery at the hip bone landmark (153.2 V/m), and the popliteal artery at the knee landmark (231.3 V/m). The highest *E_tan_* value which was found in the popliteal artery at the knee landmark was approximately 58% higher than that in the gel of the ASTM phantom.

## Discussion

In this study, we compared SAR and *E_tan_* in the ASTM phantom with those in the AustinMan model at four landmarks and evaluated whether the RF exposure in a simplified geometry with a homogeneous medium is representative of the exposure in the human body in a clinical setting. The ratio of the peak SAR value in the 10 cm trajectory to the averaged SAR value in the ASTM phantom (2.45) was comparable to that of previous studies. Amjad et al. (2005) quantified the SAR distribution at 64 MHz with two different conductivities: 0.27 and 0.6 S/m. The ratio of their local to whole-phantom SAR values were 2.5 and 2, respectively. Additionally, this ratio is in line with values reported by MR testing companies when performing RF-safety testing in ASTM phantom (Song et al., 2018).

Some parts of the human model were subject to high SAR across different landmarks. For example, simulations at both the brain and the heart landmarks showed high SAR values in the common carotid arteries, the subclavian arteries, and the innominate artery. This is likely due to the length of the coil which covered those three arteries at both the brain and the heart landmarks. A shorter coil may result in a smaller exposed body volume, and the *SAR_raw_* and E_tan_ values in those tissues may be lower. Nevertheless, tissues outside of the coil can still have high SAR due to eddy currents induced in the body as shown in the heart, hip bone, and knee landmarks (Figure 3). Thus, it is important to consider the possible effect of different coil geometrical and electrical characteristics when evaluating medical device safety.

This study focused on the safety assessment at 64 MHz (1.5 T MRI system). The scanners used in a clinical setting can have a static field up to 7 T. Therefore, further analysis may be needed to evaluate RF safety in the 3 and 7 T environments.

The *SAR_raw_* values were proportional to the *E_tan_* values in all the blood vessel trajectories that we evaluated. The popliteal artery at the knee landmark showed high *SAR_raw_* values up to 76.2 W/kg and its averaged *E_tan_* value was 154 V/m. The iliac artery at the hip bone landmark also showed high SAR values up to 68.9 W/kg and its averaged *E_tan_* value was 112 V/m. The ascending aorta and the femoral artery had mean *SAR_raw_* less than 2 W/kg and their averaged *E_tan_* values were less than 50 V/m.

However, the volume-averaged SAR in the patient-left brachial artery at the heart landmark was not in agreement with *E_tan_* because the high *SAR_raw_* was washed out by averaging over the surrounding tissues. This example shows that volume averaging is not always a proper surrogate for *E_tan_* and substantiates the need for *SAR_raw_* or *E_tan_* as metrics for safety assessment.

The length of the device also plays a key role in the quantification of heating. For electrically short devices (Volakis, 2007) with a length shorter than $\frac{\lambda }{4\pi }$ of the RF wavelength in tissues, the local background SAR at the tip of the implant can be used as surrogate metric for worst-case heating. Thus, in this scenario, *in vivo* heating may be estimated by simply scaling the background SAR with respect to *in vivo* local SAR without the need for performing a full thermal simulation with a stent. On the other hand, for a longer stent such as those used for peripheral arteries, this approach may not be valid since *E_tan_* magnitude and phase contributions along the path of the device become more significant. In these cases, both the amplitude and phase of *E_tan_* may need to be taken into account (Park et al., 2007), and full electromagnetic and thermal simulations may be needed with the device in place as per intended use.

We have shown the SAR and *E_tan_* results of the ASTM model. The phantom has a homogeneous medium with a simple rectangular structure whereas the human body consists of complex structures and various dielectric properties. The underestimation of SAR and *E_tan_* values of the ASTM phantom may be due to the simple and homogenous structure.

This study highlighted the need of detailed anatomical modeling. We have not performed a detailed modeling of the stent structure which is one of the limitations of this study. Santoro et al. (2012) have modeled the geometry of a coronary stent at a 7.0 T MRI with finite element method (FEM) in a homogeneous phantom. Modeling an actual stent with the intricate whole-body anatomical human model is a hurdle with approaches based on FDTD due to the current limitations of computational resources. On the other hand, detailed anatomical models such as AustinMan are currently not available in FEM-based platforms. We envision that an improvement of computational resources and a development of FEM-based anatomical models may enable to overcome our current limitations.

In this study, the tissue properties of blood vessels modeled were limited to electrical conductivity, permittivity, and mass density. The *in vivo* blood vessels have blood perfusion and flow which have significant effect on reducing the RF induced heating. Moore et al. (2014) have incorporated heat sink due to both blood perfusion and skin blood perfusion in their models. Gross (2016) have shown that the flow reduces the temperature rise of a stent along with the surrounding medium significantly. Scandling (2016) reported the flow rate similar to the one of the iliac artery reduced the temperature by 4°C compared to the model without the flow. Elder (2013) also showed temperature reduction with different flow rates. Incorporating the blood perfusion and the different blood flow rate will enable more robust assessment in RF safety.

There is another possible scenario where SAR alone might not be sufficient to measure heating. The vascular tree spreads out all over the body as shown in Figure 4. Stents can be implanted in multiple orientations depending on the intended use. In these cases, *E_tan_* may be more appropriate to use as a metric for safety assessment as SAR does not incorporate information on the angle of the electric field. Thus, further investigation with implantable devices placed at different angles in different tissues may be needed to assure the accuracy of the current safety assessment method.

This study focused on SAR and *E_tan_* results in the ASTM phantom and the AustinMan model. Thermal simulations were not included and it is another limitation of the study. It is expected that investigating the translation to temperature rise will provide better understanding in SAR and *E_tan_* values in each model.

Superficial tissues have higher exposure compared to deep tissues. For example, at the knee landmark, high SAR values were observed in the vessels in the ankle area. The vascular territories in this area tend to be close to the surface of the body (skin). The ankle area is one common placement for cosmetic tattoos where skin burns can occur during MRI (Ross and Matava, 2011). The detailed surrounding tissue conditions must be considered when modeling for safety evaluation.

For fine structures like skin, sub-millimeter modeling might be necessary to accurately estimate the electric field and potential heating. Currently, only the head has been modeled using a sub-millimeter spatial resolution (Iacono et al., 2015). In addition to a fine resolution, physiological responses of tissues may need to be included in the model to increase the accuracy of the prediction. Continued advancement of realistic computational modeling is necessary to assure the safety of performing MRI scans on patients with medical devices.

## Conclusion

The simulation results in this study suggest that ASTM-based testing may underestimate *in vivo* values for the vascular regions in the human body. Computational modeling with high-resolution whole-body numerical models, when properly validated, represents an important tool to evaluate accurate RF exposure in blood vessels. Also, safety assessment may need to be tailored to the physical characteristic of the medical device and its position in the human body to ensure patient safety during an MRI scan.

## Author Contributions

LA, SR, and MI conceived the project and provided overall direction. KF designed the study, performed the computational modeling, and analyzed the data. EL designed the RF coil model. EL and MI assisted computational modeling setup. KF wrote the manuscript under the supervision of MI. All authors reviewed the manuscript.

## Disclaimer

The mention of commercial products, their sources, or their use in connection with material reported herein is not to be construed as either an actual or implied endorsement of such products by the Department of Health and Human Services.

## Conflict of Interest Statement

The authors declare that the research was conducted in the absence of any commercial or financial relationships that could be construed as a potential conflict of interest.

## Supplementary Material

FIGURE S1 The blood vessel 3D SAR maps at the brain, heart, hip bone, and knee landmarks are shown. The color scale was set such that the maximum was twice the peak SAR calculated in the whole ASTM phantom model (9.0, 8.7, and 8.2 W/kg for *SAR_raw_*, *SAR*_1*g*_, and *SAR*_10*g*_, respectively) to optimize the visualization of the peaks across all the models.
