# Supplementary figures and images for: Radio-Frequency Safety Assessment of Stents in Blood Vessels During Magnetic Resonance Imaging
Source: Front Physiol. 2018 Oct 22;9:1439. doi: 10.3389/fphys.2018.01439 (PMC6232906; doi:10.3389/fphys.2018.01439)

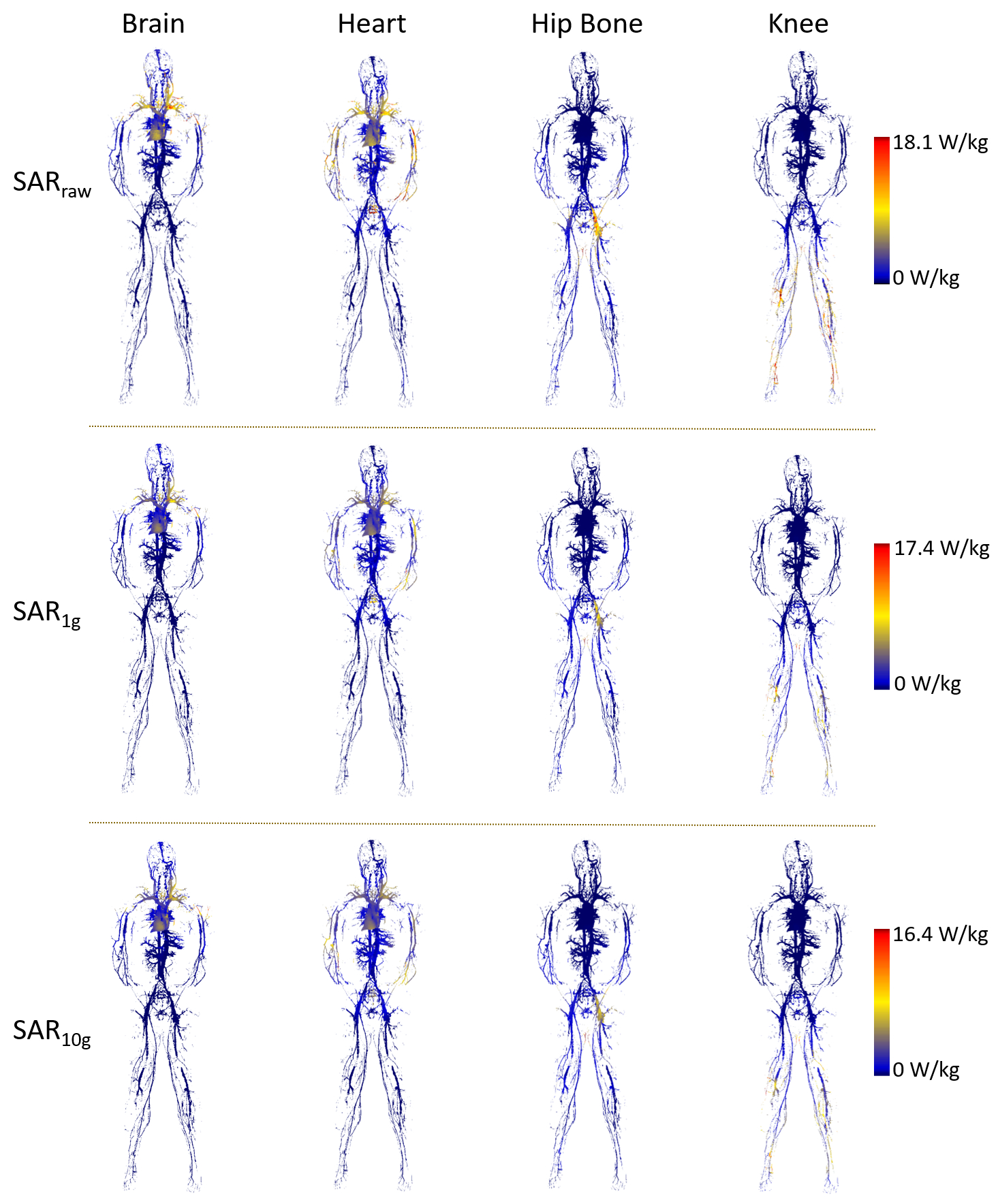

Supplement: FIGURE S1 — The blood vessel 3D SAR maps at the brain, heart, hip bone, and knee landmarks are shown. The color scale was set such that the maximum was twice the peak SAR calculated in the whole ASTM phantom model (9.0, 8.7, and 8.2 W/kg for SARraw, SAR1g, and SAR10g, respectively) to optimize the visualization of the peaks across all the models. [file Image_1.JPEG]
